# Advancement in Paper-Based Electrochemical Biosensing and Emerging Diagnostic Methods

**DOI:** 10.3390/bios13070689

**Published:** 2023-06-28

**Authors:** Stephen Rathinaraj Benjamin, Fábio de Lima, Valter Aragão do Nascimento, Geanne Matos de Andrade, Reinaldo Barreto Oriá

**Affiliations:** 1 Drug Research and Development Center (NPDM), Federal University of Cearà, Fortaleza 60430-270, CE, Brazil; gmatos@ufc.br; 2 Department of Physiology and Pharmacology, Faculty of Medicine, Federal University of Cearà, Fortaleza 60430-270, CE, Brazil; 3 Post Graduate Program in Health and Development in the Central-West Region of Brazil, Federal University of Mato Grosso do Sul UFMS, Campo Grande 79070-900, MS, Brazil; valter.aragao@ufms.br; 4 Laboratory of the Biology of Tissue Healing, Ontogeny and Nutrition, Department of Morphology, Institute of Biomedicine, School of Medicine, Federal University of Cearà, Fortaleza 60430-270, CE, Brazil; oria@ufc.br

**Keywords:** paper-based devices, electrochemical sensors, environmental analysis, clinical analysis, food analysis

## Abstract

The utilization of electrochemical detection techniques in paper-based analytical devices (PADs) has revolutionized point-of-care (POC) testing, enabling the precise and discerning measurement of a diverse array of (bio)chemical analytes. The application of electrochemical sensing and paper as a suitable substrate for point-of-care testing platforms has led to the emergence of electrochemical paper-based analytical devices (ePADs). The inherent advantages of these modified paper-based analytical devices have gained significant recognition in the POC field. In response, electrochemical biosensors assembled from paper-based materials have shown great promise for enhancing sensitivity and improving their range of use. In addition, paper-based platforms have numerous advantageous characteristics, including the self-sufficient conveyance of liquids, reduced resistance, minimal fabrication cost, and environmental friendliness. This study seeks to provide a concise summary of the present state and uses of ePADs with insightful commentary on their practicality in the field. Future developments in ePADs biosensors include developing novel paper-based systems, improving system performance with a novel biocatalyst, and combining the biosensor system with other cutting-edge tools such as machine learning and 3D printing.

## 1. Introduction

Early detection of diseases can have a significant impact on patient outcomes. It can enhance the precision and efficiency of disease diagnosis if practical examples of the successful usage of diagnostic tools in developing nations are provided. Additionally, providing more information on the types of diseases that can be detected early using these tools can help readers understand the importance of early detection. It is important to consider the limitations or challenges associated with implementing these diagnostic tools in remote areas with limited access to medical care. This will provide a more balanced perspective on the potential implications of this technology and help readers understand the complexities of improving healthcare in emerging nations.

PADs have emerged as a promising new method for implementing point-of-care applications in the past few decades. The potential of PADs as inexpensive disposable sensors has garnered considerable interest owing to their excellent surface-to-volume ratios, cost-effectiveness, portability, and user-friendly design. Moreover, it is noteworthy that PADs exhibit a remarkable degree of sensitivity and selectivity towards diverse analytes, thereby enabling the detection of small amounts of molecules. This characteristic renders them highly appropriate for a broad spectrum of applications. PADs are advantageous due to their potential for long-term monitoring and rapid response times. Their simplicity and portability make them ideal for medical applications, such as the detection of biomarkers for diagnostics and therapeutic monitoring. Since Dungchai et al. first described electrochemical detection PADs (ePADs), they have gained popularity because of their simplicity, low power consumption, low detection limits, and easy quantification capabilities [1].

Most commonly, PADs are detected utilizing colorimetric and electrochemical techniques. Colorimetric techniques use indicators that exhibit a change in color in the occurrence of an analyte, while electrochemical methods measure the current produced by a reaction between the analyte and a working electrode. Colorimetric methods are usually less expensive and simpler to operate than electrochemical techniques, and they can detect a wide variety of analytes. Colorimetric paper-based diagnostics have an equipment-free read-out, but raw blood obscures a colorimetric response which has motivated diverse efforts to develop blood sample processing techniques [2].

However, they are not as sensitive as electrochemical methods and unsuitable for continuous monitoring. On the other hand, electrochemical methods offer higher sensitivity and the ability to monitor analytes continuously, but they are more expensive and require more complex equipment. Colorimetric paper-based devices are advantageous in ease of measurement and interpretation, but they have limitations due to their sensitivity to interference in colored matrices (such as blood). However, ePADs showed great sensitivity and enabled the detection of analytes in both turbid and colored mixtures, consequently expanding the scope of ePADs’ potential uses. Several research studies have explored the development of electrochemical, portable PADs for the analysis of substances across diverse domains, including clinical [3], environmental [4], and food safety [5].

**Figure 1:**
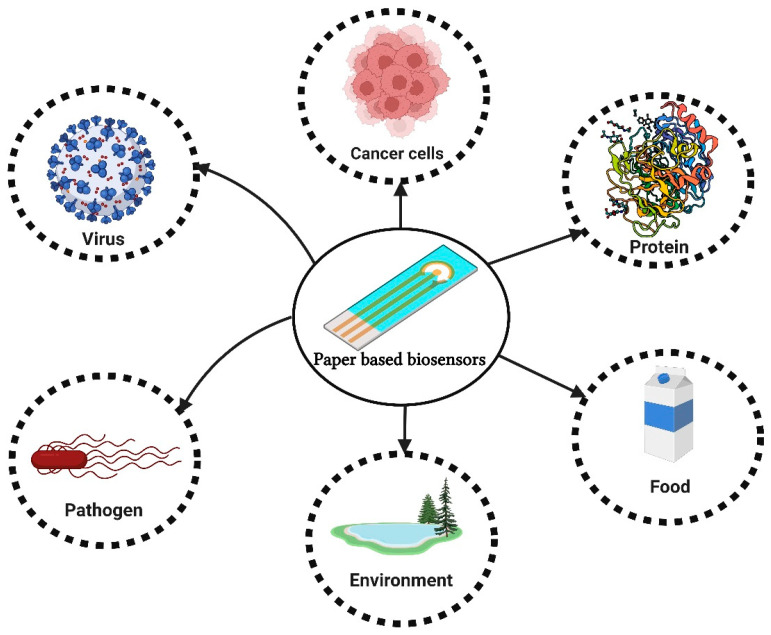
Schematic illustration of applications of the paper-based biosensors.

This review aims to provide a comprehensive overview of the emerging advances in ePADs, including their design and functionality, and to evaluate their potential use in various fields (Figure 1). The review also examines current limitations and future possibilities for the use of ePADs. It also provides an assessment of the current challenges and opportunities ePADs present and potential applications for the future. Furthermore, the prospective utilities of ePADs in clinical, environmental, and food analysis are evaluated.

### Paper-Based Biosensors

Paper-based sensors are analytical instruments that employ paper as a base material to identify and measure diverse analytes. The sensors are low price, portable, and user-friendly, rendering them suitable for implementation in settings with limited resources, such as emerging economies or field environments [6,7]. The enhancement of PADs’ effectiveness can be attained by integrating advanced detection methodologies and cutting-edge technology. Moreover, they provide advantages such as a short time to analyze a sample and the use of a small amount of sample (few μL) that can be analyzed. Hence, these aforementioned devices exhibit potential as substitutes for the conventional POC devices presently employed. Paper is employed as a material for the substrate in conjunction with two or three electrodes to serve as the electrode area in ePADs.

The employment of paper-based electrochemical sensors faces two primary challenges. Firstly, the performance of these sensors is subject to significant variation due to the diverse features of the substrate, including porous structure, network, and surface [8]. Additionally, the complex structure and specific regulations in small-scale production contribute to the uncertainty in reproducibility and accuracy, which are crucial considerations. Second, paper-based sensors’ surfaces are required to be developed and adjusted for selective detection, and their performance cannot be regulated on a broad scale [9]. The PADs are compatible with different detection modes, such as colorimetric, fluorescence, electrochemical, surface-enhanced Raman spectroscopy (SERS), and magnetic detection.

A paper-based electrochemical biosensor (PEB) is a type of biosensor that utilizes a paper substrate as a platform for electrochemical sensing. The modification of the paper substrate involves incorporating materials with conductivity, including carbon nanotubes (CNT) or gold nanoparticles (AuNPs), which serve as electrodes for analyte detection. The utilization of printed electrodes on PEBs presents a number of notable benefits in comparison to conventional electrochemical biosensors. These advantages include affordability, disposability, and user-friendliness. One of the most common applications of PEBs in medical diagnostics is for detecting glucose levels in the blood [10]. In general, PEBs exhibit potential as a viable medium for creating cost-effective and easily transportable diagnostic instruments for the management of diabetes and other diseases that necessitate regular monitoring of blood biomarkers.

## 2. Design and Fabrication of Paper

The potential use of paper as a biosensor material is highly attractive owing to its flexibility, wide accessibility, economical nature, and hydrophilic properties. Its surface can readily be changed or shaped and has potent adsorptive capabilities for biomolecules and nanoparticles (NPs). Cellulose, the most common biopolymer, is a linear chain comprising several glucose units and the main ingredient of most paper kinds. Cellulose is a biodegradable fiber that is strong, non-absorbent, and water-insoluble [11].

The advantages of the paper include its non-toxic properties, easy disposal, and biodegradability. One of the primary advantages of cellulose is its porosity, which facilitates the capillary transport of solutions. Consequently, cellulose has the potential to function as a self-sufficient microfluidic pumping mechanism, avoiding the requirement for additional pumps. Paper’s inherent features (such as biocompatibility, porosity, flatness, and capillary forces) allow for novel developments in various applications. The affordability, ease of manufacture and operation, portability, and disposability of paper-based analytical equipment are all significant factors in their widespread use. The material’s adaptability renders it highly suitable for analysis decentralization, as demonstrated by its application in lateral and vertical flow devices [12], single or complex components, and its ease of integration with other flat materials such as tape. Additionally, this material can be manipulated through cutting, pressing, folding, or printing.

### 2.1. Paper Types Used in Electroanalytical Devices

Paper-based analytical instruments typically use cellulose fiber content-based materials or membranes made from nitrocellulose, two of the many paper types now available. Whatman^®^ filter papers are widely used in the fields of chromatography and filtering because they are made from cellulose fiber (cellulosic paper). The most popular grade, no. 1, has moderate retention and flow rates. Most paper-based analytical devices are fabricated utilizing materials based on cellulose fibers or nitrocellulose membranes (Table 1).

Paper-based analytical strips are cheaper to produce, more accessible to dispose of (they may be burnt), and need fewer operations (particularly, reagent-free devices). Due to the porous nature of filter paper, chemicals could be preloaded into the strips [19]. Paper substrates often utilized for PAD fabrication include paper filters, blotting paper, and chromatography sheets of paper. Paper is a flexible, inexpensive, and widely available material. The development of PADs has extensively used Whatman type 1 chromatographic paper and the filter paper from Whatman no. 1. The materials that make up each paper generally consist almost entirely of cellulose (more than 98%). The advantages of using Whatman grade 1 chromatography are less expensive paper cost, high flow rate, consistent thickness, and good hygroscopic and wicking capacities. Both Whatman grade 4 papers for chromatography (pore size of 20–25 µM) and nitrocellulose (NC) paper have been utilized as substrates for protein immobilization due to their robust protein-binding capabilities, which are attributed to charge–charge connections and weak secondary factors, respectively. The paper-based sensors were fabricated utilizing diverse paper types, including chromatography paper from grade 3, paper towels, Whatman P81, and office paper. Although many different paper substrates are already in use, researchers are always on the lookout for more sustainable options.

Microfluidic channels, lateral flow [20], dipsticks, and origami designs are just a few of the many configurations for the instrument. The integration of origami-based paper sensors presents potential advantages in detecting volatile components, as it enables the absorption of vapor within the paper pores, followed by direct detection. Origami’s resistance to leaching and the coffee impact after reacting with analytes keeps the sensing assay’s rapid reaction times intact. Despite using various paper substrates in the construction of PADs, researchers continue to seek out substrates with distinctive attributes or locally produced through environmentally sustainable methods. Several methods are available for fabricating fluidic channels or assay zones on PADs, including inkjet printing [21], screen printing [22], wax printing [23], photolithography, plasma treatment, flexographic printing, wax dip, and laser cutting [24,25].

### 2.2. Fabrication of Paper-Based Electrodes

Several common methods exist for integrating sensing materials into paper substrates for paper-based biosensors. The choice of a suitable methodology depends on both the specific application scenario and the attributes of the sensing material. Some standard methods for integrating sensing materials into paper substrates include drop casting, inkjet [26], screen printing, spray coating, and electrodeposition methods [27,28].

### 2.3. Electrochemical Biosensors

Electrochemical biosensors are equipped with an identification component and electronic transducer, which facilitate the detection of highly sensitive analytes in human body fluids. Moreover, the high suitability of these devices for POC diagnostics is attributed to their portability, wearability, and implantability. These sensors include potentiometric, voltammetric, amperometric, photoelectrochemical, organic electrochemical transistors, and electro chemiluminescent sensors [29].

The electrochemical biosensor is a representative sensing apparatus that transduces biochemical occurrences into electrical signals. The current sensor design employs an electrode as a primary component, which functions as a reliable material for immobilizing biomolecules and promotes the transfer of electrons. Electrochemical biosensors present several benefits compared to traditional laboratory analytical methods, such as high-pressure liquid and gas chromatography combined with mass spectrometry. The primary attributes of these instruments encompass their capacity for mobility, ease of use, cost-effectiveness, quick analysis duration, elevated severity, and specificity within complex matrices [30,31]. Numerous nanoparticles with broad surface areas improve loading capacity and reactant mass transfer for high analytical sensitivity [32]. Recent years have seen an increase in the manufacturing of these biosensors, connected to the widespread utilization of nanomaterials such as graphene, its compounds, and nanocomposites (e.g., graphene oxides (GO) and CNT) [33,34].

Graphene, a hexagonal lattice structure, has garnered significant attention recently. Graphene has found utility in diverse biosensor uses, particularly in the field of electrochemical sensors [35]. Graphene has identical fundamental physicochemical properties to graphite and carbon nanotubes, alongside its extensive surface area and numerous functional sites. Graphene’s exceptional electronic and mechanical characteristics make it suitable for label-free biosensors [36]. Nanomaterials used as electrodes or facilitating matrices should have assisted electrocatalytic properties, excellent electron movement capacity, and remarkable biocompatibility with capturing biomolecules for signal amplification. Electrochemical techniques can be employed to incorporate nanomaterials into paper and microfluidic biosensors, thereby enabling the identification of biomolecules at the POC.

### 2.4. Improvement of ePAD Detection Performance by Nanomaterials

The employing of nanomaterials as electrode materials has the potential to expedite electron transfer and enable the effective loading of biomolecular explores and electrochemically reactive molecules [32,37]. Therefore, nanomaterials can be used to fabricate electrodes for ePADs, and they can also be used to modify as-fabricated electrodes with nanomaterials to enhance their detection sensitivity. For improving the detection efficacy of the screen-printed carbon electrodes (SPCEs) in the ePADs, various nanoparticles were used, including noble metal (gold, platinum, and silver) nanoparticles, metallic oxides, and silica nanoparticles. Nanoparticles facilitate the rapid transfer of electrons among the transducer and analyte while also serving as covalent anchoring points for a variety of biorecognition probes. A recent study demonstrated that the carbon electrode in ePADs can effectively alter AuNPs, enabling electrochemically thiolated DNA probes to be constructed. The utilization of AuNPs in the fabrication of a carbon electrode on a paper substrate leads to an increase in both anodic and cathodic currents, as evidenced by enhanced charge, conductivity, and electrically charged area of the surface. This is attributed to the conductive properties of the AuNPs, which function as a conductive layer. It is observed that the ePADs are highly sensitive to the detection of microRNA 155 with a LOD of 33.8 nM [38]. Furthermore, the paper-based carbon electrode enhanced with AuNPs displays remarkable stability after being stored for 60 days in the dark. Paper-based EC strips were produced by Cinti et al. [39], who utilized a drop-casting method to deposit AuNPs onto a carbon electrode made from paper, with the aim of detecting both single- and double-stranded DNA.

The utilization of AuNPs in immobilizing MB-tagged triplex-forming oligonucleotides (TFO) at the electrode surface and improving charge transfer kinetics is attributed to their distinctive characteristics, including favorable electrical properties, robust adsorption ability, facile attachment by covalent bonds to thiolated compounds, and elevated surface-to-volume ratio. Carbon nanotubes (CNTs), both single-walled and multi-walled, have been widely utilized as transduction components in electrochemical (EC) biosensors owing to their remarkable conductivity to electricity, expansive surface area, and varied surface-chemical properties. In addition, the utilization of paper coated with carbon nanotubes facilitates the production of nanostructures that possess porosity and a substantial surface area. Electrodes made from CNTs on paper have attracted considerable attention for use in ePADs. Valentine et al. [8] deposited MWCNT-Nafion onto pre-cut sensing areas of chromatography paper using an ethanol suspension of the nanocomposites. The impact of paper pore size on the final MWCNT network and subsequent electrochemical sensing of glucose was investigated in this study. Tran et al. [40] fabricated patterned single-walled carbon nanotube electrodes by vacuum filtering a layer of SWCNT onto a nitrocellulose (NC) membrane, which took approximately 15 min. The electrodes displayed notable conductive properties, using a mean resistance measuring below 100/sq, and exhibited remarkable mechanical durability when subjected to repeated bending assessments. AuNPs have the potential to be utilized for the development of non-enzymatic glucose ePADs through direct deposition onto single-walled carbon nanotube electrodes.

### 2.5. Designing and Fabricating 3D Paper Devices

The present methodology enables the construction of three-dimensional paper-based apparatuses through the utilization of folding, cutting, and lamination techniques applied to paper sheets, resulting in intricate geometric configurations. The paper devices are filled with active materials such as enzymes, dyes, and nanomaterials to create functional, low-cost, and sustainable devices. These devices are easy to produce, cost-effective, and eco-friendly. They have many applications, including medical diagnostics and sensing [41,42,43]. Recently, Guan et al. [44] employed glue adhesion to build a paper-based, three-dimensional analytical device with separate, specialized layers for separating plasma and detecting fibrinogen in blood samples and detecting linear values from 127.0 mg/dL to 508.0 mg/dL. In addition, the analytical device based on 3D origami does not require electricity or specialized instruments. It has the ability to detect HIV type 1p24, an infectious disease, at low levels as 0.03 ng/mL [43]. The paper-based analytical device utilizing 3D origami has the capability to detect human immunoglobulin G (HIgG) at levels as low as 0.01 ng/mL [17].

Teengam et al. [45] have devised a biosensor with high sensitivity for detecting *M. tuberculosis*, demonstrating its potential for rapid and low-cost tuberculosis diagnosis. The biosensor utilizes the covalent immobilization of the pyrrolidinyl peptide nucleic acid (PPNA) on the 3D paper-based device, allowing for detecting *M. tuberculosis* nucleotides. The findings revealed that the biosensor demonstrated a range of linearity of 2 to 200 nM and LOD value of 1.24 nM, indicating the potential for precise and sensitive detection of nucleotides associated with *M. tuberculosis*.

### 2.6. Wearable Sensors

Paper-based electrochemical sensors that are wearable are a specific type of sensor that employs electrochemical techniques for sensing and paper as the substrate material. These sensors have gained popularity recently owing to their affordability, user-friendliness, and mobility [46]. The paper used in these sensors is typically functionalized with conductive materials, including graphene, carbon nanotubes, or silver nanoparticles, to enable electrochemical sensing. The sensors are also coated with a layer of enzyme or antibody, which allows for detecting specific analytes [47]. The flexibility and conformability of wearable paper-based electrochemical sensors render them advantageous for monitoring biological signals and health indicators on the skin. They can be integrated into wearable devices, such as smartwatches or fitness trackers, to continuously monitor biomarkers such as glucose, lactate, or cortisol. Additionally, using paper as a substrate makes them environmentally friendly and biodegradable. Wearable paper-based electrochemical sensors have a broad spectrum of potential applications in diverse fields such as temperature [48], pressure [49], light [50], humidity and respiration [13], and healthcare (monitoring of biomarkers, diabetes, or cardiovascular disease).

## 3. Applications

Paper-based electrochemical analytical devices have been widely utilized in various fields due to their inherent benefits such as rapidity, affordability, simplicity, and disposability. The initial research efforts primarily focused on medical implementations and worldwide health concerns. However, various other domains surfaced, such as clinical (Table 2), food security, environmental, and forensic investigations.

### 3.1. Clinical Analysis

The most relevant biomolecules and biomarkers (glucose, hydrogen peroxide [65], dopamine, uric acid, ascorbic acid, urea, and cholesterol) were determined using ePADs [66,67]. The utilization of paper-based sensors has surfaced as a propitious framework for POC testing and other immediate diagnostic procedures owing to their economical nature, adaptability, and inherent self-pumping capability. For instance, Mazzaracchio et al. [68] employed nanomaterials, specifically carbon black (CB) and AuNPs, to fabricate an electrochemical sensor on the paper substrate for the purpose of detecting iron ions in serum samples. The biosensor showed a high detection of 0.05 mg/L and a linear range of values of 10 mg/L.

Yamaoka et al. [69] constructed a novel PAD for glucose detection. The potentiometric sensor for glucose detection was developed using a combination of a CMOS chip and fluidic channels derived from paper, resulting in a cost-effective solution. This study used chromatography paper and silicone resin as fluidic channels. The channel consisted of a filter layer (for filtering a sample) and an enzyme layer (for reacting with an enzyme). This electrochemical sensor has been enhanced by adding a CMOS chip enabling the measurement of glucose levels from 0.5 to 10 mM.

Glucose concentrations in urine can be easily and semi-quantitatively monitored using a novel electrochemical glucose sensor [70]. The system is composed of a sensing strip that is disposable and paper-based, as well as an amplifier circuit that is simple and includes a visual display. Five enzyme-activated electrodes were used on the paper strip. The electrodes were connected to an indicator system that activated the illumination of a light-emitting diode (LED) upon sensing glucose concentrations descending within a pre-established range. The study employed graphene and Ag-doped silica nanoporous SBA-16 electrodes in ePADs for the purpose of detecting L-cysteine. The detection was carried out using a commercial glucose meter [71] that showed a low detection limit (0.02 µM) and a linear range (0.1 to 250 µM). Recently, Dincer et al. [65] have proposed a cost-effective, portable, and self-calibrating method for the in situ tracking of H_2_O_2_ levels in simulated exhaled breath, with continuous and instantaneous monitoring capabilities. The developed portable system employs easily manufacturable ePADs that feature an analog electrode configuration. These ePADs are equipped with a Prussian blue accomplished carbon electrode that enables the sensing of H_2_O_2_, and a carbon black electrode that effectively eliminates noise signals. The results present a linear measuring range of 5–320 µM H_2_O_2_ exhibiting a sensitivity of 0.19 nA µM^−1^ mm^−2^.

#### Detection of Nucleic Acid

The growing attention towards the identification of particular sequences of DNA is a positive sign for the advancement of disease diagnosis. Henry et al. [46] have recently created a label-free 3D-ePAD electrochemical DNA biosensor for the purpose of detecting *M. tuberculosis* in blood samples that have been amplified by PCR. The linearity range of 2–200 nM and a detection limit of 1.24 nM were determined under optimal conditions. Chailapakul et al. [72] have introduced a new biosensor composed of electrochemical DNAs on paper, which can be utilized for the swift detection of High-Risk HPV type 16. The biosensor incorporates an electrode that has been modified with an anthraquinone-peptide nucleic acid (PNA) probe and a graphene-PANI/SPCE electrode. The designed DNA detector demonstrated excellent selectivity for non-complementary sequences. The integration of this sensor to identify HPV-DNA type 16 with LOD of 2.3 nm and linearity range of 10–200 nM acquired from cancer cell lines was successful.

### 3.2. Detection of Viruses

#### COVID-19 Detection Using Paper-Based Biosensors

Paper-based systems have the potential to serve a broad range of purposes, ranging from basic screening to precise quantification. These systems may utilize paper test strips, electrochemical biosensors, lateral flow systems, and computational methods to achieve their intended objectives [73,74,75]. Graphene/carbon electrodes were screen-printed on paper substrates and used as impedance sensors to detect coronavirus as part of a measurement of nasopharyngeal fluid samples [76]. Recently, there have been potential applications of nanobiosensors for monitoring SARS-CoV-2 in environmental conditions [75,77].

**Figure 2:**
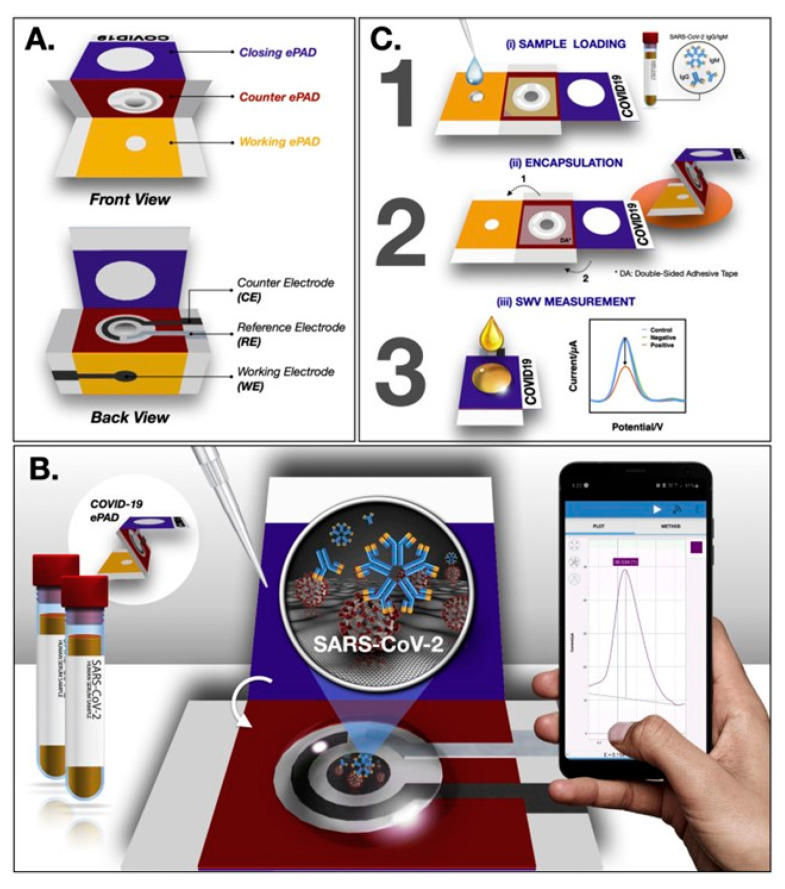
COVID-19 ePAD schematic diagram showing (**A**) device components, (**B**) detection principle, and (**C**) detection technique, Reproduced with permission from [75].

Yakoh et al. [75] developed an additional ePAD that facilitates expedited and precise identification of SARS-CoV-2 antibodies. The present platform was constructed utilizing three sets of electrodes arranged in a configuration reminiscent of the art of origami. The paper sheets were individually equipped with electrodes, namely, the working ePAD, counter ePAD, and closing ePAD, and subsequently integrated into the final design through folding. Significantly, this setup can reduce or eliminate environmental exposure to potentially harmful fluids. The immobilization of S protein RBD onto highly hydrophilic paper incorporated with graphene oxide (GO) was achieved through EDC/NHS chemical reactions. Subsequently, the functional ePAD was employed to print the WE. The ePAD in a closed state was subjected to a solution comprising the redox investigation [Fe(CN)_6_]^3/4^ for the purpose of electrochemical detection. The kinetics of the redox process were determined based on the immunocomplex development involving the collected immobilized SARS-CoV-2 S protein and protein immunoglobulins, which resulted in the conversion of the measured signal. IgG and IgM detection analytical performance was enhanced, with LOD values of 0.96, 0.14 ng/mL, and a linearity range of 1–3000 ng/mL (Figure 2). The analytical results obtained were in concurrence with the ELISA gold standard utilized in commercial environments. Electrochemical techniques have exhibited greater responsiveness and lower detection limits in the identification of antibodies. However, these benefits are accompanied by the need for additional equipment for signal collection and read-out. On the other hand, LFAs have demonstrated a lower level of complexity in usage and are more cost-effective.

Pardee et al. [78] successfully integrated Cas9 cleavage behavior with the amplification of nucleic acid techniques, such as NASBA and CRISPR-based isothermal amplification (NASBACC). The described integration was employed to distinguish closely associated RNA Zika virus variants in an artificial environment with exactitude at the single base level, utilizing toehold switch RNA sensors and a desiccated, paper-based platform. The RNA strand of the Zika virus undergoes a process of unfolding the toehold, which subsequently exposes a binding site for ribosomes. This event leads to the transformation of protein molecules responsible for inducing a change in color. Zika RNA samples are detected using toehold switches, isothermal amplification, and a disk-based drying system. Furthermore, a polydiacetylene (PDA) colorimetric biosensor was devised for point-of-care diagnosis, capable of detecting pH1N1 viruses with high infectivity with influenza A viruses through chromatic modifications [79].

Jokerst et al. developed a study that found a new method for detecting COVID-19 using M^pro^, which plays a crucial role in the virus’s life cycle. They used gold colloids and substrate to quickly detect M^pro^, causing a color change in the nanoparticles in less than 10 min. This test is effective in detecting M^pro^ in breath condensate with <10 nM concentration and has no false positives in negative COVID cases [80].

### 3.3. Biomarkers for Disease Detection

#### 3.3.1. Detection of Cardiovascular Biomarkers

Cardiovascular diseases are the primary cause of mortality worldwide and are associated with a growing need for healthcare resources. It comprises a range of conditions that impact the heart and blood vessels. The development of this disease is influenced by a number of risk factors, such as hypertension, tobacco use, hyperlipidemia, diabetes mellitus, sedentary lifestyle, excessive body weight, and a familial predisposition to cardiovascular disease. Clinicians, environmental factors, and healthcare providers worldwide face difficult early diagnosis and intervention challenges. Biomarker detection using an impedimetric immunosensor devised for detecting cardiac troponin I at the point of care. Mathur et al. [81] developed a PAD framework that uses MWCNTs functionalized with carboxyl groups and antibodies to detect cardiac troponin I. The designed sensor exhibits a response time of under one minute and possesses a sensitivity of 1.85 m/ng/mL and a LOD of 0.05 ng/mL. Troponin I, C-reactive protein (CRP), and procalcitonin are three important CVD biomarkers that can be determined simultaneously using an ePAD and a label-free immunoassay technique [82]. The ePADs were stencil-printed with graphene oxide-modified carbon electrodes to immobilize antibodies against the target biomarkers in serum samples.

#### 3.3.2. Detection of Cancer Biomarkers

Pereira et al. [46] constructed a PAD modified with an aptamer (cancer biomarker) for colorimetric analysis of osteopontin. Then, (mercaptopropyl) methyl dimetoxisilane was used to chemically modify cellulose paper to bind the thiolated aptamer, a biological detection layer. The LOD of the paper-based aptasensor was below 5 ng/mL, demonstrating its high sensitivity. Wang et al. [58] developed an electrochemical aptasensor constructed from paper for monitoring CEA and NSE in the early identification of lung cancer. Specific cancer biomarkers may be more easily and specifically detected using label-free electrochemical immunoassay technology. The screen and wax printing processes resulted in a three-electrode system and microchannels. Nanocomposites of amino-functional graphene (NH_2_-G)/thionin (THI)/gold nanoparticles (AuNPs) and Prussian blue (PB)/poly(3,4-ethylene dioxythiophene) (PEDOT)/AuNPs were used to modify the working electrodes and boost the aptasensor’s sensitivity. The aptasensor can detect concentrations of CEA between 0.01 and 500 ng mL^−1^, with detection limits of 2 pg mL^−1^ and 10 pg mL^−1^, respectively. In human serum, CEA and NSE values of 5 and 15 ng mL^−1^, respectively, are used for diagnosing lung cancer.

#### 3.3.3. Detection of Other Molecules/Macromolecules

The detection of biomolecules depends on the biochemical and physiological activities of small biomolecules such as nucleic acids, enzymes, and hormones. These functions include the transcription of genetic details, regulation of biological action, and catalysis of reactions within cells. The challenges associated with the advancement of biological molecule detection technology persist. Gel electrophoresis, Western blotting, and polymerase chain reaction (PCR) are widely used techniques in the field of biomolecular analysis. Electrochemical detection possesses the ability to overcome the limitations of traditional techniques while also offering advantages such as rapid accessibility, economical feasibility, and superior sensitivity and selectivity.

Recently, a new electrochemical sensing technology has been developed that can be used for DNA diagnostics. For instance, Cinti et al. [39] developed a paper-based strip to identify both single and double-stranded DNA in serum samples after PCR amplification of a dsDNA sequence related to HIV was successfully applied. The recognizing probes were double-stranded triplex-forming oligonucleotides, including methylene blue (MB), based on paper-based AuNPs-SPE platforms.

The origami paper-based analytical device (oPAD) employs a mechanical valve in the form of a pop-up framework, enabling it to achieve a novel multi-mode differential pulse voltage (DPV)/supercapacitor to amplify the signal read-out structure. This represents a significant advancement in the field. The detection of adenosine 5′-triphosphate (ATP) is achieved through the initiation of glucose oxidase (GOx) reactions by the aforementioned device [83]. The DPV read-out mode can generate an electrochemical signal by means of enzymatic oxidation of glucose through residual GOx-DNA2 present in the sensing zone. This can be achieved by a straightforward alteration in the orientation of the pop-up structure. The proposed self-sustaining organic photodiode array (oPAD) facilitated the precise examination of ATP within a linear value of 10–5000 nM, exhibiting detection limits of 3 nM and 1.4 nM, correspondingly.

### 3.4. Detection of Genes

Recently, an electrochemical immunosensor has been developed that utilizes screen-printed carbon nanotube-polydimethylsiloxane electrodes for the label-free identification of multiple avian influenza virus antigens [84]. There has also been the development of ePADs suitable for the voltammetric detection of miRNA biomarkers (miRNA-155, miRNA-21) using rGO or moS_2_ nanosheets decorated with AuNPs [85]. The identification of a complementary miRNA target sequence was accomplished through the utilization of differential pulse voltammetry (DPV) within a redox solution. This solution contained a thiol-linked synthetic DNA probe that was immobilized on the working electrode through the reduction of its potential.

#### Viral RNA Testing

Kumar et al. [86] designed a CRISPR diagnostic structure to identify the essential concern variants in SARS-CoV-2’s genome. Lateral flow paper strip chemistry detected the S gene mutation N501Y (present in various SARS-CoV-2 variant lineages) within one hour. The CORDS fluorescence and paper strip technologies can quickly and sensitively identify SARS-CoV-2 mutations in DNA targets reverse-transcribed from RNA. The lateral flow strip of the RT-CORDS has a LOD of 10^−16^ M [87].

Alafeef et al. [88] developed an electrochemical paper sensor chip capable of targeting the N-gene with excellent specificity and sensitivity, with an assay time of less than 5 min compared to conventional biochemical sensing methods. This sensor is made by modifying chromatography paper with graphene nanoplatelets and depositing gold microelectrodes using electron beam deposition. Lastly, these electrodes were coated with gold nanoparticles that had been embedded with antisense oligonucleotides that had been specifically designed for the N gene. In the COVID-19 study, the sensor detected a voltage change after nasopharyngeal swab samples entered with exceptional accuracy (near 100%), indicating a LOD of 6.9 copies/L. In addition, MERS-CoV and SARS-CoV were not detected by the proposed method due to its high specificity. Several paper-based SERS analytical platforms with exceptional performance in specificity, sensitivity [89], accuracy, and stability were identified for multiple analytes such as adenine [90], *S. typhi* [91], catechol [92], sulfur dioxide [93] environmental pollutants [94], and molecular contaminants [95] using SERS detection schemes, coupled with an easy assay procedure and rapid response.

### 3.5. Point-of-Care ePADs for Neurotransmitter Monitoring

Neurotransmitters are significant biological substances implicated in numerous nervous system disorders (NSDs). ePADs are capable of detecting neurotransmitters from very small sample sizes, allowing for quick and accurate diagnosis of neurotransmitter-related diseases [34]. Furthermore, these ePADs are highly sensitive and able to detect even trace amounts of neurotransmitters, making them an invaluable tool for preclinical studies. The results of these ePADs are also reproducible, meaning that the same results can be obtained by repeating the experiment. This makes them highly reliable and accurate for diagnosing and studying neurotransmitter-related diseases.

Settu et al. [96] fabricated a screen-printed electrode using graphene material in order to create an ePAD for the purpose of detecting dopamine (DA). The electrode surface was modified with PEDOT:PSS/RGO to produce a screen-printed graphene electrode (SPGNE). Dopamine was detected linearly from 0.0125 to 0.1 mM by the paper-based SPGNE biosensor, with a sensitivity of 24.9 μA mM^−1^.The design and development of Janus-ePADs represents an innovative approach to producing advanced multiplexed sensing apparatuses that possess the extensive potential for detecting norepinephrine and serotonin, 5-hydroxytryptamine, 5-HT, concurrently through electrochemical means, as well as detecting *p*-aminophenol [97].

### 3.6. Environmental and Food Analysis

The utilization of ePADs has been widespread in the identification of diverse environmental contaminants, including heavy-metal anions, explosives, neurotoxins, pesticides, volatile organic substances, and other minute molecules. Present advancements in sensing techniques based on paper have been made for the detection of pollutants [98]. Based on an origami paper sensor, pesticide aerosols can be selectively detected with an optoelectronic nose [99]. Arduni et al. [100] have exhibited exceptional characteristics and emphasized the necessity of a tool with the ability to identify various categories of pesticides over smart environmental detection.

The 3D origami design with incorporated multi-paper electrochemical biosensors shows great potential for detecting pesticides based on enzyme inhibition, due to the flexibility of the origami method. This paper-based technology employs two electrodes printed with several paper filter pads to provide multiple analyses for detecting various pesticides through various enzymes immobilized on a lab-on-a-chip. Colozza et al. have developed wearable electronic devices that can rapidly detect mustard agents, such as 2-chloroethyl sulfide, as released in the aerosol form of chemical weapons. This is a significant step forward in chemical warfare defense technology [101]. The biosensor under consideration is reagent-free and requires only filter paper for support, owing to the preloading of all chemicals onto the origami sheets. The inhibition of choline oxidase by mustard gas chemicals was observed through the measurement of hydrogen peroxide production through a screen-printed electrode that had undergone treatment with a carbon black and Prussian blue nanocomposite. This wearable sensor system can be integrated with military uniforms, drones, and portable devices in the event of a terrorist attack in order to detect mustard agents with accuracy.

Recently, the functional composite paper sensors have demonstrated electrocatalytic behavior under moderate conditions upon electrochemical reduction. The composite paper exhibits cost-effectiveness, conductive and electrocatalytic capabilities for electrochemical sensing. The composite’s repeatability and scalability are superior to paper-based sensors made through surface modification (i.e., printing method). Hydroquinone, chlorophenol, and nitrophenol were detected at 0.045, 0.093, and 0.571 mg L^−1^, respectively [102].

Additionally, paper electrodes coated with gold nanoparticles and graphene nanosheets were fabricated for nitrite detection in water and milk samples. The calibration curve for nitrite detection has a LOD value of 0.1 μM and a linear range of 0.3–720 μM [103]. The measurement of Cr speciation is a commonly performed task utilizing paper-based colorimetry. Recently, Wang et al. [104] carried out PAD with colorimetric/electrochemical dual read-outs to detect total and hexavalent chromium (Cr(VI)). The LOD of Cr(VI) and total Cr was 0.01 mg L^−1^, and the linearity ranges of 0.05–3.0 mg L^−1^ and 0.2–3.0 mg L^−1^ were identified.

**Figure 3:**
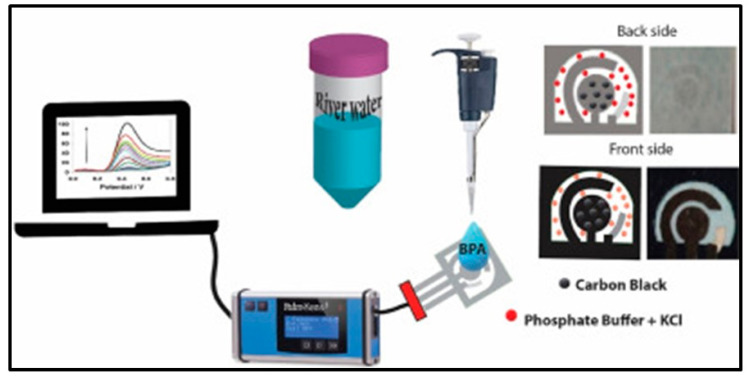
Detection of bisphenol A without reagents using a paper-based electrochemical sensor. Reproduced with permission from [105].

Bisphenol A (BPA) is essential in manufacturing epoxy resin, polycarbonate, and unsaturated polyester resins. In recent years, however, BPA has been implicated as an endocrine disruptor. Arduini et al. [105] presented an electrochemical device for BPA detection that only requires the inclusion of the sample, made possible by printing the electrodes on filter paper, storing the reagent materials in the filter paper, and treating the sample with the reagents. Square wave voltammetry is an electrochemical method optimized for a LOD of 0.03 μM over two linear bands (0.1 to 0.9 μM; 1 to 50 μM) in water samples (Figure 3).

#### Humidity Sensors

Typically, the optimal range of humidity that generates a comfortable sensation in the human body is within 40 to 70%, provided that the temperature is adequate. A humidity sensor measures breath RH to track breathing patterns and provide health data in real time since released breath is considerably more humid than ingested air. A novel printed graphene humidity and respiration sensor has been successfully fabricated and validated on paper substrates by inkjet printing. The sensors showed great sensing capabilities for RH levels across 10% and 70%, with a sensitivity value of 0.03 pF/RH%.

Furthermore, this demonstrates the promise of constructed paper for monitoring the respiration rates of humans, an application that might greatly benefit from the quick reaction and recovery times of paper sensors (5 s) [18]. The identification of aflatoxin B1 through the utilization of a chitosan/MWCNT film-based immunosensor that is disposable in nature. The immunosensor was subjected to modification with anti-AFB1 for the purpose of the study. The impedimetric immunosensor showed excellent sensitivity with a LOD of 0.62 ng mL^−1^ and a wide linear range of 1–30 ng mL^−1^. Additionally, the sensing platform proved to be efficacious in the identification of aflatoxin B1 in maize flour samples through the utilization of extraction solutions while exhibiting a high selectivity in the presence of other mycotoxins [106].

### 3.7. Foodborne Pathogens

Global population growth increases the need for more food production. The importance of food quality monitoring methods to combat foodborne diseases increases as food production increases. The demand for cost-effective and highly responsive sensors capable of detecting pathogenic bacteria in foodborne applications has been on the rise, particularly in the food industry. The most dangerous foodborne bacteria include *Campylobacter*, *Salmonella*, *E. coli O157:H7*, *S. aureus*, and *L. monocytogenes.* Most PADs that detect infectious bacteria use qualitative or semi-quantitative colorimetric or fluorometric detection methods.

**Figure 4:**
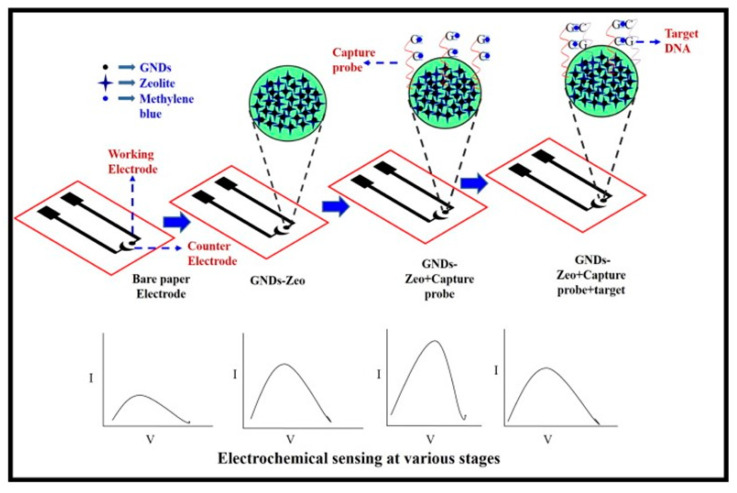
ePAD-based matrices employing Graphene nano dots and Zeolite for the detection of *S. aureus* in fruit samples, reproduced with permission from [109].

The integration of electrochemical, electrochemiluminescence, and photoelectrochemical detection methods into paper-based electrodes can be achieved through the application of conductive materials via printing techniques [107]. The authors Bharadwaj et al. [108] presented a new and cost-effective electrochemical biosensor that utilizes paper as a substrate for detecting *S. aureus*. The biosensor employs SWCNT antibody conjugates to enable rapid detection of the pathogen. This method detects spiked milk samples at 13 CFU mL^−1^ in 30 min. Mathur et al. [109] employed paper-based Geno interfaces with graphene nanodots and zeolites to detect *S. aureus* in fruit samples at 0.1 nM (Figure 4). Chen et al. [110] developed the paper-based NIR photoelectrochemical sensing method for ultrasensitive identification of *E. coli O157:H7* using a flexible conductive paper electrode with a LOD value of 2 CFU/mL.

Recently, a potentiometric immunosensor based on disposable paper for real-time foodborne pathogen detection *S. typhimurium* [111]. Antigen–antibody conjugation blocks the ionic flux, which is correlated with *S. typhimurium* concentrations based on the potential shift caused by the blocking effect. The proposed method has been successfully demonstrated on apple juice samples, proving its capability for real-time applications with an assay time of less than 1 h and a low LOD of 5 cells/mL. In a recent study, DNA-Au/Pt bimetallic nanoclusters with peroxidase-like catalytic activity were used to detect *S. aureus* bacteria in food samples [112]. Furthermore, the optical sensor had a linearity value of 10^8^–10^2^ CFU/mL with a LOD value of 80 CFU/mL.

### 3.8. Paper-Based Electrochemical Microfluidic Devices

During the last decade, there have been significant advancements in microfluidic paper-based analytical devices (μPADs). They are often used to hold chemical reagents and convey fluid samples for colorimetric and electrochemical determination of various analytes. Cellulose paper-based μPADs can process liquid quantities from 0.1 to 100 μL. These devices feature fluidic channels that are measured in millimeters. They are manufactured by assembling papers, adhesive films, and cut paper to fabricate 3D channels. Microfluidic devices can be assembled using stacking, origami, and lamination [113].

The use of μPADs are preferred for point-of-need applications requiring rapid analysis at low cost. Nevertheless, μPADs offer additional advantages apart from their physical durability. The hydrophilic and porous nature of cellulose facilitates capillary action, which enables flow without the requirement of external power sources or pumps. This phenomenon is attributed to the cohesive and adhesive forces [114,115]. The van der Waals force results from the intermolecular attraction between fluid molecules at the interface of liquid and air. On the other hand, the surface tension force is a consequence of the interface between the liquid and fiber. Furthermore, the utilization of PADs enables cost-effective production of the apparatus that is user-friendly and disposable due to its non-trouser nature, while also yielding prompt results. Microfluidics enables improved decision-making by facilitating fluid migration without the need for external force application. The aforementioned characteristics of PADs render them an ideal POCT platform in that analysis is conducted close to the patient’s bedside, utilizing a limited sample size to furnish prompt, consequential outcomes that can enhance clinical decision-making [116].

Adhesion and wetting depend on paper matrix surface qualities, which may be quantified by the fluid angle of contact at the liquid–fiber interface. Additionally, the comprehension of the interplay among the constituents from the wicking mechanism of a μPAD is restricted. The hydrophilic hydroxyl groups on the cellulose fibers may interact with different regions of protein molecules, depending on the solvent composition [117]. It has the potential to impact the process of protein folding. Furthermore, the hydrophobic barrier present at the interface has the potential to impede the movement of the active reagent that is bound to the analyte, leading to a modification of its rate of transportation.

#### Integration of Electrochemical Cells in Paper-Based Analytical Devices

The integration of an electrochemical cell into PADs adds an additional layer of detection capability. Electrochemical cells can detect changes in electrical properties that result from the chemical reaction on the paper substrate, allowing for quantitative measurements of analytes. Electrochemical cells are specified, and the working electrode’s surface is often changed to increase selectivity for the target biomarker. Wax-printing procedures are often used in producing devices because they allow for the precise patterning of the paper’s various regions. The electrochemical cell is located in a hydrophilic detecting region, and the appropriate solutions are drop-cast directly into this region to detect the electrical conductivity of the cell.

In addition, an electrochemical origami-based PAD was created for detecting pre-albumin (PAB) and CRP, both are employed in the diagnosis of gastrointestinal cancer. The hydrophobic region and the electrodes were made using printing and wax printing. The DPV methodology was employed for evaluating the reactivity of the CRP and PAB. Additionally, PAB and CRP electrochemical responses were linear within concentration ranges of 10 pg/mL to 1 g/mL and 5 pg/mL to 1 g/mL, respectively. PAB’s LOD was 10 pg/mL, and CRP’s was 5 pg/mL [118].

## 4. Carbon Paper

Carbon materials are extensively utilized in bioelectrochemical applications, encompassing a range of materials such as graphite, carbon paper, and carbon felt. Carbon materials have been shown to exhibit different morphological patterns and structural characteristics. These electrode materials are considered to be the most beneficial type of application of bioelectrochemistry because they enable microorganisms to conduct electron transfer processes at high rates [119]. Recently, carbon paper-based electrochemical biosensors have been developed for photosynthetic herbicide detection based on thylakoid membranes [120].

This study demonstrates the stability of thylakoid interactions with carbon paper, suggesting that the thylakoids are supported by weak non-covalent interactions (e.g., van der Waals, stacking (π–π), and hydrogen bonds), which in turn stabilize the thylakoid–carbon paper interaction. The biosensor can generate photocurrent levels of approximately 14 μA/cm^2^ without redox mediators. In this work, the possibility of using carbon fiber-based composite paper as an economically viable and high-surface framework for the fabrication of biosensors. The application of PCA/SWNT ink onto paper was accelerated by means of wax printing and vacuum filtration, resulting in the development of hydrophilic and well-defined channels, all without the utilization of masks or stencils. Human serum albumin (HSA) is detected with a LOD of 1 pM, demonstrating its ability to be detected quantitatively and selectively [11].

### Limitations

There are a number of factors that affect the performance of an ePAD: 1. Target analyte concentration: The incubation period of a paper-based biosensor can be affected by the amount of the target analyte. Higher concentrations of the analyte may require shorter incubation times, while lower concentrations may require longer incubation times. 2. Sensing material: The sensitivity of the sensing material can also impact the incubation time of a paper-based biosensor. More-sensitive sensing materials may require shorter incubation times, while less-sensitive materials may require longer incubation times. 3. Sample matrix: The matrix of the sample can affect the incubation time of a paper-based biosensor. Samples with high viscosity or complexity may require longer incubation times to allow for proper interaction with the sensing material. 4. Environmental factors: Environmental factors, such as temperature and humidity, can also affect the incubation time of a paper-based biosensor. Humidity can also affect the stability and activity of the sensing material, which can impact the incubation time. 5. Detection method: The choice of detection method can also impact the incubation time of a paper-based biosensor. Some detection methods, such as colorimetric methods, may require longer incubation times to allow for proper color development, while other methods, such as electrochemical methods, may require shorter incubation times. Overall, many factors can affect the incubation time of a paper-based biosensor, and it may need to be optimized for accurate results. The incubation time of a paper-based biosensor should be determined by the manufacturer’s instructions as well as the application and sample type. 6. Matrix effect: In sensor technology, the phenomenon of nonspecific adsorption of molecules in the matrix can have a significant impact on the sensor’s response. This can result in a change in the sensor’s output, leading to difficulty in accurately detecting and measuring the target analyte. 7. Stability: The storage conditions of paper-based electrochemical sensors are important for maintaining their stability. This involves regular testing of the sensor’s physical, chemical, and electrical properties. Additionally, temperature, humidity, light, and mechanical stress impact the stability of the sensors.

## 5. Conclusions and Perspectives

In recent years, electrochemical sensors that utilize paper as a substrate have emerged as promising tools for detecting various analytes. These sensors have demonstrated efficacy in diverse areas such as medicine, agriculture, and military applications. The enhancement of the analytical performance of the system can be achieved through the integration of electrodes that are supported on a paper substrate with either bioreceptors or nanomaterials. This process can lead to an increase in the system’s sensitivity, selectivity, and adaptability. Since numerous functions may be merged and automated on simplified platforms, future research should focus on developing multiplexed devices with 2D, 3D, or origami designs to enhance device performance and reduce analysis times. It is also of significant interest to improve ePAD detection by developing inks with better electrochemical characteristics for electrode deposition.

Recent advancements in ePAD technology show great potential for improving gadgets, from the laboratory to the marketplace. The success of portable, user-friendly ePADs is essential for their widespread adoption. Similarly, ePADs used for POC diagnostics or in locations with few resources must be reliable and long-lasting tools. Despite the clear advantages of ePADs, most of the research covered in this article concentrates on the manufacture of devices rather than their final function in applications or the validation of analytical methods. This gap in knowledge highlights the tremendous untapped potential for further study in this area.

Electroanalytical characteristics, including mass and charge transmission, diffusion, electrode poisoning, adsorption, and their impacts owing to the manufacturing process, paper type, electrode material, and ink concentration, have received insufficient attention in basic research. In the next phase of ePAD research, such investigations enable a critical evaluation of methodologies and materials over “test and works”. The electrochemical behavior of the electrodes should be addressed while selecting paper, materials, and manufacturing procedures to ensure both sensitivity and accuracy in detecting the specific analyte. Additionally, future work will need to test the sensor’s stability (and shelf life), which is crucial for its use in real samples and commercialization.

Future efforts will allow the study of ePADs to expand beyond academia, drawing the attention of the commercial and technological fields, demonstrating its importance to society in early disease diagnosis, disseminating diagnostic methodologies to areas with low adequate health and sanitation care, and enhancing public safety and health decision-making. Paper’s unique properties, including its porosity and its ability to be folded, provide electrochemical devices based on paper with unique features, including the preconcentration capacity that enables reagent-free, very sensitive tests. Recently, the paper’s porosity has been employed as a reactor to synthesize nanomaterials, resulting in a functionalized decorated paper that may be utilized in printing the electrochemical cell.

Furthermore, these devices have broken ground in removing the restriction that the target analyte can only be detected in a liquid sample. The industrial community’s focus on this equipment category has led to the creation and widespread adoption of plastic-free alternatives. The concerns of repeatability and robustness in humid settings are of great significance in this approach. These concerns require prompt attention in order to facilitate market entry, provide feasible diagnostic equipment to the community, and enhance the multibillion-dollar paper diagnostic industry. However, innovative biosensor systems may benefit from the incorporation of cutting-edge technology such as machine learning [67] and 3D printing methods. The improved sensitivity of ePADs is made possible by the exact construction of a miniaturized system, which is now possible due to 3D printing. Globally, the ePADs could effectively replace existing methods of continuous monitoring.

## Figures and Tables

**Table 1:** Summary of different types of paper substrates and their characteristics.

Type of Paper Substrate	Sensing Material	Fabrication Method	RH Range (%)	Sensitivity	Reference
Filter paper (Whatman 4)	Carbon black (CB) and Reduced graphene oxide (rGO)	Coating and drying	33–95%	0.7 (33−75%) 1.5 (75−95%)	[13]
Printing paper	Polyimide	Laser writing	0–90%	-	[14]
Cellulose filter paper	Cobalt chloride	Soaking and drying	11–98%	-	[15]
A4 printing paper	A4 printing paper	Facile pasting	7.2–91%	-	[16]
A4 porous paper (metallic pearl)	Graphite and silver nanoparticles	Screen printing and pencil drawing technique	70–95%	0.0564%	[17]
Printing paper	Glycidyl trimethyl Ammonium chloride (EPTAC)	Screen printing	11–95%	1.59 (54 RH%) 63.7 (95 RH%)	[15]
Metalized paper (aluminum-coated paper)	Polymeric layer	Laser ablation	2–85%	18.9 fF/%RH	[16]
Cellulose paper	Carbon nanotube and Polydimethy siloxane composite	Screen printing	30–95%	0.375 pF/RH% (30–70%) 8.24 pF/RH% (70–95%)	[17]
Glossy paper	Graphene printing ink	Inkjet printing	40–70%	0.03 pF/RH%	[18]

**Table 2:** Summary of paper-based biosensors used in electrochemical immunosensor and aptasensor detection.

S.NO	Target	Material	Detection Methods	Linear Range	LOD	References
Electrochemical immunosensor						
1.	CA 15.3	Ag-RGO/CysA-Au NPs	ChA	15–125 U/mL	15 U/mL	[51]
2.	CA 125	rGO/Thi/AuNPs	CV, DPV	0.1~200 U/mL	0.01 U/mL	[36]
3.	Spike protein of SARS-CoV-2	CNC-SPGE	DPV	0.1 pg/mL to 500 ng/mL	2.0 fg/mL	[52]
4.	Human IFN-γ	PANI/G	EIS	5–1000 pg/mL	3.4 pg/mL	[53]
5.	Glycoprotein	SiO_2_@Au/dsDNA/CeO_2_	DPV	1 pg/mL–1000 ng/mL	0.87 pg/mL	[54]
6.	Alpha-fetoprotein	Ni-Co MOF	DPV	1–200 ng/mL	0.3 ng/mL	[55]
7.	Cardiac troponin I	MXene	EIS	5–100 ng/mL	0.58 ng/mL	[56]
8.	5-hydroxy-l-tryptophan	Graphene nanoribbons	SWV	25–1000 μmol/L	7.6 μmol/L	[57]
Aptasensor						
9.	Carcinoembryonic antigen	NG-THI-AuNPs-PB-PEDOT	DPV	0.01–500 ng/mL	2 pg/mL	[58]
10.	17β-Estradiol	Amino redox graphene/thionine/streptavidin-modified gold nanoparticles/chitosan	DPV	10 pg/mL to 100 ng/mL	10 pg/mL	[59]
11.	Kanamycin	Graphene	Potentiometry	0.05–30 pM	0.05 pM	[60]
12.	Breast cancer cells (MCF-7)	Paper-based aptasensor and gold nanoparticles	CV, DPV and EIS	20 to 1 × 10^6^ cells mL^−1^	7 cells mL^−1^	[61]
13.	Programmed death-ligand 1	Amino carbon nanotubes/methylene blue	CV, DPV	10 pg/mL–2.5 ng/mL	10 pg/mL	[62]
14.	Oxytetracycline	Multiwall carbon nanotubes, gold nanoparticles, reduced graphene oxide and chitosan	CV, DPV and EIS	1.00–540 nM	10 nM	[63]
15.	SARS-CoV-2	Carbon nanofibers and gold nanoparticles	EIS	0.01–64 nM	7.0 pM	[64]

## Data Availability

Not applicable.
